# Phenotypic and Genotypic Characteristics of Uropathogenic *Escherichia coli* Isolates from Kenya

**DOI:** 10.1089/mdr.2020.0432

**Published:** 2022-01-13

**Authors:** Catherine Wawira Muriuki, Lilian Adhiambo Ogonda, Cecilia Kyanya, Daniel Matano, Clement Masakhwe, Erick Odoyo, Lillian Musila

**Affiliations:** ^1^ Department of Biomedical Science and Technology, School of Biological and Physical Science, Maseno University, Maseno, Kenya.; ^2^ Department of Emerging Infectious Diseases, United States Army Medical Research Directorate-Africa, Kenya/Kenya Medical Research Institute, Nairobi, Kenya.

**Keywords:** phenotypic, genotypic, ESBL, pAmpC, uropathogenic *Escherichia coli* (UPEC), antimicrobial resistance, urinary tract infections

## Abstract

***Introduction:*** Uropathogenic *Escherichia coli* (UPECs) are a significant cause of urinary tract infections (UTIs). In Kenya, UTIs are typically treated with β-lactam antibiotics without antibiotic susceptibility testing, which could accelerate antibiotic resistance among UPEC strains.

***Aim:*** This study determined the occurrence of UPEC producing extended-spectrum β-lactamases (ESBLs), the genes conferring resistance to β-lactams, and the phylogenetic groups associated with ESBLs in Kenyan UPECs.

***Methodology:*** Ninety-five UPEC isolates from six Kenyan hospitals were tested for ESBL and plasmid-mediated AmpC β-lactamase (pAmpC) production by combined disk diffusion and disk approximation tests, respectively. Real-time and conventional polymerase chain reactions (PCRs) were used to detect three ESBL and six pAmpC genes, respectively, and phylogenetic groups were assigned by a quadruplex PCR method.

***Results:*** Twenty-four percent UPEC isolates were ESBL producers with *bla*_CTX-M_ (95.6%), *bla*_TEM_ (95.6%), and *bla*_SHV_ (21.7%) genes detected. Sixteen isolates had *bla*_CTX-M/TEM_, whereas five had *bla*_TEM/CTX-M/SHV_. A total of 5/23 ESBLs were cefoxitin resistant, but no AmpC genes were detected. The UPECs belonged predominantly to phylogenetic groups B2 (31/95; 32.6%) and D (30/95; 31.6%), while groups B2 and A had the most ESBL producers.

***Conclusions:*** β-Lactam antibiotics have reduced utility for treating UTIs as a quarter of UPECs were ESBL producing. Single or multiple ESBL genes were present in UPECs, belonging primarily to phylogenetic groups B2 and A.

## Introduction

Globally, an estimated 150 million people contract urinary tract infections (UTIs) annually and account for most adult outpatient visits.^1^ The prevalence of UTIs ranges from 6% to 37% in developing nations.^2^ UTIs result from the ascent of bacteria from the periurethral area to the urethra, bladder, and upper urinary tract.^3^ Colonization of the periurethral area with uropathogenic bacteria is a critical factor in causing UTIs.^3^ Women are more likely to suffer from UTIs than males due to their shorter urethral distance and proximity of the urethral tract to the anus.^4^ Among women, the prevalence is higher in pregnant women.^5^ A recent study in Kenya among pregnant women indicated a prevalence of 15.7% UTIs.^6^ The severity of UTI depends upon the virulence of bacteria and the host's susceptibility.^5^ Uropathogenic *Escherichia coli* (UPEC) is the primary etiological agent of UTIs.^7^ β-Lactam antibiotics are the main class of drugs used to treat hospital and community-acquired UPEC infections.^8^ The emergence and spread of bacterial resistance to β-lactam leading to treatment failure and recurrent infections are of clinical concern.^9^ In *E. coli*, the main mechanism of resistance to β-lactam antibiotics^10,11^ is the production of extended-spectrum β-lactamases (ESBLs) and plasmid-mediated β-lactamases (pAmpCs).

*E. coli* ESBL producers' occurrence is geographically variable and is correlated with the overuse of antibiotics.^12^ ESBL producers have been reported in several African countries: Egypt, Morocco, Tunisia, Senegal, South Africa, and Nigeria, with prevalence rates ranging from 5% to 44.3%.^12^ Studies of ESBL producers among *E. coli* isolates from fecal samples in Kenya show prevalence rates ranging from 39% in malnourished children^13^ to 3.4% in healthy adults.^14^ Few studies have focused on determining the prevalence among UPEC strains, but both ESBL- and pAmpC-producing bacteria have been reported in UPECs in Kenya.^15^ The genes conferring resistance to ESBLs often coexist on the same plasmid with genes conferring resistance to other antibiotics.^9,11^ These plasmids facilitate the horizontal transmission of resistance to multiple antibiotics used to treat bacterial infections.^16^ Therefore, ESBL production by UPEC can hinder the effective management of UTIs.^17^ Multidrug-resistant (MDR) UPEC limits therapeutic options, leading to increased morbidity and health care costs.^12,18^ The ESBL genes that have been associated with *E. coli* isolated from enteric, environmental, and wound samples in Kenya are predominantly *bla*_CTX-M_ and *bla*_TEM_ genes.^13,15,19^

The AmpC β-lactamases confer resistance to penicillins, cephems, and monobactams, but not carbapenems.^9,11^ These enzymes are not inhibited by β-lactamase inhibitors such as clavulanate, tazobactam, and sulbactam,^9,11^ but are inhibited by boronic acid and cloxacillin.^10^ In *E. coli*, AmpC β-lactamases are encoded by genes located either on the chromosome or plasmids.^20^ Plasmid-mediated AmpC β-lactamases (pAmpCs) have origins from chromosomally encoded AmpCs of multiple genera within the Enterobacteriaceae family and display structural and functional similarities.^21^ The most commonly detected plasmid-mediated AmpC is the CMY type,^15^ of which there are 64 plasmid-mediated variants.^20^ Plasmid-mediated AmpC β-lactamase production in bacteria is associated with multidrug resistance due to the carriage of resistance determinants for other antibiotic classes on the same plasmids.^11^ Chromosomally mediated AmpC genes do not cause resistance to cephalosporins unless they are overproduced.^22^

β-Lactams are often used for the empirical treatment of UTI. Despite the changing epidemiology of ESBL genes, there are insufficient data on the occurrence of β-lactam genes in *E. coli* isolates from UTI patients in Kenya. Therefore, continuous surveillance to provide knowledge of genetic determinants of resistance to commonly prescribed antibiotics in different regions is essential. Surveillance outcomes can guide the selection of effective antibiotic therapy and implementation of infection control strategies to minimize the spread of resistant bacteria. Determination of *E. coli* phylogenetic groups is of epidemiological significance as isolates belonging predominantly to phylogenetic group B2 are associated with virulence of extraintestinal strains.^23^ Therefore, this study aimed to determine the occurrence of ESBL producers, ESBL genes (*bla*_TEM_, *bla*_SHV_, and *bla*_CTX-M_), and pAmpC genes (*bla*_MOX_, *bla*_CIT_, *bla*_DHA_, *bla*_ACC_, *bla*_EBC_, and *bla*_FOX_) and the phylogenetic groups of UPEC in isolates from patients with UTI from several hospitals in Kenya.

## Methods

### Bacterial isolation and culture

The Department of Emerging Infectious Diseases (DEID) of the United States Army Medical Research Directorate-Africa, Kenya (USAMRD-A, Kenya), based in the Kenya Medical Research Institute (KEMRI), Nairobi, has been conducting antimicrobial resistance surveillance in Kenya since April 2015 (KEMRI SERU#2767/WRAIR IRB#2089). This study utilized 95 archived UPEC isolates collected between April 2015 and August 2018 in that study. Urine specimens had been collected from individuals older than 2 months with UTI symptoms, at both in- and outpatient departments, who gave informed consent. For individuals <18 years old, the parent or guardian provided informed consent before participating in the parent protocol. These participants were enrolled from six public health care facilities in five counties: Kisumu, Kericho, Kilifi, Kisii, and Nairobi. One of the following three methods was used to obtained urine samples: midstream clean-catch urine collection, pediatric urine bags from infants and young children, or sterile needles and syringes to collect fresh urine from existing catheter tubing. The urine samples were transferred in boric acid and transported at room temperature within 48 hours to the Centre for Microbiology Research laboratories in KEMRI for testing.

The urine samples were cultured in CLED and MacConkey media to detect bacterial pathogens and bacteria were identified on the VITEK 2 (bioMerieux) platform. Antimicrobial susceptibility testing (AST) was performed using the GN83 and AST-XN05 VITEK 2 (bioMerieux) panels, which collectively test a panel of 27 antibiotics (cefoxitin, ceftazidime, cefotaxime, cefuroxime, cefixime, ceftriaxone, ciprofloxacin, clindamycin, aztreonam, azithromycin, tetracycline, oxacillin, gentamicin, erythromycin, levofloxacin, clarithromycin, minocycline, imipenem, amoxicillin/clavulanic acid, ampicillin/sulbactam, amikacin, trimethoprim, colistin, tigecycline, chloramphenicol, and moxifloxacin). Results were interpreted using the Clinical and Laboratory Standards Institute (CLSI) guidelines.^24^ Isolate identification and antibiotic susceptibility results were recorded in the study database. MDR isolates were defined as nonsusceptible to at least one antimicrobial agent in three or more antimicrobial classes.^25^ Extensively drug-resistant isolates were defined as bacterial isolates susceptible to only one or two antimicrobial classes.^25^ Pandrug-resistant isolates were defined as nonsusceptible to all agents in all antimicrobial classes.^25^ The bacterial isolates were archived in glycerol stocks at −80°C for future studies. All isolates of UPEC collected from April 2015 to August 2018 and viable on subculture on Muller–Hinton agar (MHA; Becton Dickinson) were included in the study.

### Phenotypic confirmatory test for ESBL production

#### Combination Disk Diffusion Test

Based on the VITEK 2 results, the UPEC isolates that showed a minimum inhibition concentration (MIC) ≥16 μg/mL for ceftazidime, according to CLSI guidelines, were subjected to the combination disk diffusion test (CDDT) as an ESBL confirmatory test. In brief, a ceftazidime 30-μg disk (Becton Dickinson) and combination ceftazidime+clavulanic acid 30 + 10-μg disk (Becton Dickinson) were placed 60 mm away from each other and incubated at 37°C for 24 hours. A difference in zone size of >5 mm between the ceftazidime+clavulanic acid disk and the ceftazidime disk is confirmatory for ESBL-producing strains as per CLSI guidelines because ESBL producers are inhibited by clavulanic acid. Ceftazidime (a third-generation cephalosporin) resistance is indicative of an ESBL-producing isolate. *E. coli* ATCC 25922 was the ESBL-negative control, and *Klebsiella pneumoniae* ATCC 700603 was the ESBL-positive control.

### AmpC screening using the disk diffusion method

The UPEC isolates that were found to be positive for ESBL production were also screened for presumptive AmpC β-lactamase production by the disk diffusion method using a cefoxitin disk of 30 μg (Becton Dickinson). AmpC producers, unlike ESBL isolates producing other enzymes, are resistant to cephamycins, such as cefoxitin. In brief, a cefoxitin disk of 30 μg (Becton Dickinson) was placed at the center of a plate inoculated with the bacterial isolate and incubated at 37°C for 24 hours before reading the results. Isolates with zone diameters <18 mm, indicating resistance as per CLSI guidelines, were presumed positive for AmpC β-lactamase screening and selected for confirmatory AmpC production testing.^26,27^

### AmpC phenotypic confirmatory test

A disk approximation test was used to detect inducible AmpC production. In brief, the cefotaxime 30-μg disk and cefoxitin 30-μg disk (Becton Dickinson) were placed 20 mm apart on a plate inoculated with the test isolate and incubated overnight at 37°C. Distortion of the zone of inhibition adjacent to the cefoxitin 30-μg disk indicated the production of pAmpC. A negative result was the absence of distortion near the cefoxitin 30-μg disk.^27,28^

### Genotyping of ESBL-producing isolates

#### Detection of ESBL genes using a multiplex, real-time polymerase chain reaction

The DNA was extracted from isolates confirmed to be ESBL positive using a standard boiling method.^29^ DNA extracts were screened for the presence of *bla*_TEM_, *bla*_SHV_, and *bla*_CTX-M_ genes by fluorescent probe-based, multiplex, real-time polymerase chain reaction (PCR) using the MIC PCR instrument (Bio Molecular Systems) using published primers and probes for *bla*_TEM_, *bla*_SHV_, and *bla*_CTX-M_ (New England Biolabs).^29^ The positive controls used in this assay were in-house controls expressing the *bla*_TEM_ and *bla*_CTX-M_ genes and *E. coli* MRSN#489100 to detect *bla*_SHV_ genes. A DNA-free PCR and nuclease-free water taken through the DNA extraction process were the negative controls.

#### Multiplex PCR for detection of pAmpC β-lactamase genes

Multiplex PCR for detection of pAmpC genes (*bla*_MOX_, *bla*_CIT_, *bla*_DHA_, *bla*_ACC_, *bla*_EBC_, and *bla*_FOX_) was performed using published primers (New England Biolabs).^21^ The positive control strain used was CMY-2-positive *E. coli* (MRSN#570581), and a DNA-free PCR and nuclease-free water taken through the DNA extraction process were the negative controls.

#### Phylogenetic grouping of uropathogenic *E. coli* isolates

The new Clermont phylogenetic grouping method^30^ improves the specificity of phylogenetic assignments using the presence or absence of two or three markers to assign *E. coli* isolates into one of seven phylogenetic groups. The banding patterns observed from the PCR are used to determine the genotype and the isolate assigned to a phylogenetic group. Isolates with ambiguous groupings are tested using D or C group-specific primers (New England Biolabs).^31^ The positive control strain used was *E. coli* (ATCC 25922), and a DNA-free PCR and extracted nuclease-free water taken through the DNA extraction process were the negative controls.

DNA from the UPEC isolates was amplified by a published quadruplex PCR assay, using primers that target three markers: *chuA*, *yjaA*, and TspE4.C2.^30^ The assay also amplifies *arpA*, which acts as an international control for DNA quality and distinguishes the phylogenetic group F, formally mistaken as phylogenetic group D.^31^

### Data analysis

Data were analyzed using the Statistical Package for Social Sciences (SPSS), version 20. Descriptive statistics were used to compute the frequencies and percentages for the occurrence of ESBLs and pAmpC β-lactamases plus phylogenetic groups in UPEC isolates. The statistical significance of the distribution of ESBLs within the phylogenetic groups was determined using Fisher's exact test.

## Results

### Confirmation of ESBL and AmpC production using CLSI-approved phenotypic tests

A total of 23 (24.2%) of the 95 UPEC isolates collected between April 2015 and August 2018 screened positive for ESBL production by MIC (≥16 μg/mL) using ceftazidime (Table 1). These isolates were further confirmed to be ESBL producers by the CDDT. Five of the 23 ESBL producers were identified as cefoxitin resistant using the AmpC screening test, which is indicative of an ESBL phenotype conferred not by the typical genes, but by an AmpC gene. However, none of the five UPEC isolates showed a flattening of the inhibition zone and were classified as negative AmpC producers using the disk approximation test.

**Table 1.** Antimicrobial Susceptibility Test Results of Uropathogenic *Escherichia coli* Isolates to Ceftazidime and Cefoxitin Cephalosporins to Confirm Extended-Spectrum β-Lactamase and AmpC Production

	ESBL detection		ESBL confirmation		AmpC confirmation			
Isolate ID	A: Ceftazidime MIC > 16 μg/mL	Inter.	B: Difference in zone size > 5 mm	Inter.	C: Cefoxitin disk zone < 18 mm)	Inter.	D: Disk approximation	Inter.
UPEC 1	>16	POS	21	POS	20	NEG	NEG	NEG
UPEC 2	>16	POS	12	POS	15	POS	NEG	NEG
UPEC 3	>16	POS	14	POS	26	NEG	NEG	NEG
UPEC 4	>16	POS	14	POS	20	NEG	NEG	NEG
UPEC 5	>16	POS	14	POS	22	NEG	NEG	NEG
UPEC 6	>16	POS	17	POS	24	NEG	NEG	NEG
UPEC 7	>16	POS	15	POS	23	NEG	NEG	NEG
UPEC 8	>16	POS	17	POS	20	NEG	NEG	NEG
UPEC 9	>16	POS	17	POS	12	POS	NEG	NEG
UPEC 10	>16	POS	17	POS	20	NEG	NEG	NEG
UPEC 11	>16	POS	16	POS	22	NEG	NEG	NEG
UPEC 12	>16	POS	8	POS	20	NEG	NEG	NEG
UPEC 13	>16	POS	10	POS	17	POS	NEG	NEG
UPEC 14	>16	POS	15	POS	24	NEG	NEG	NEG
UPEC 15	>16	POS	21	POS	25	NEG	NEG	NEG
UPEC 16	>16	POS	16	POS	21	NEG	NEG	NEG
UPEC 17	>16	POS	10	POS	20	NEG	NEG	NEG
UPEC 18	>16	POS	15	POS	13	POS	NEG	NEG
UPEC 19	>16	POS	17	POS	23	NEG	NEG	NEG
UPEC 20	>16	POS	13	POS	17	POS	NEG	NEG
UPEC 21	>16	POS	13	POS	23	NEG	NEG	NEG
UPEC 22	>16	POS	19	POS	17	POS	NEG	NEG
UPEC 23	>16	POS	8	POS	23	NEG	NEG	NEG
*Klebsiella pneumoniae* (ATCC 700603)	—	—	10	POS	—	—	—	—
*E. coli* CMY-2 (MRSN#570581)	—	—	—	—	6	POS	NEG	NEG
*E. coli* (ATCC#25922)	—	—	0	NEG	22	NEG	NEG	NEG

ESBL and AmpC production were confirmed using CLSI-approved phenotypic tests. A: Ceftazidime minimum inhibitory concentration (MIC) of >16 μg/mL. B: Difference in zone size >5 mm for the ceftazidime (30 μg) + clavulanic acid (10 μg) disk compared with the ceftazidime (30 μg) disc (ESBL POS). C: AmpC screening test using the cefoxitin disc (30 μg). Inhibition zone size <18 mm, AmpC screen POS. D: Disk approximation confirmatory test for AmpC production using the cefoxitin disk (30 μg) and cefotaxime disk (30 μg).

CLSI, Clinical and Laboratory Standards Institute; ESBL, extended-spectrum β-lactamase; Inter., interpretation of the test result; MIC, minimum inhibition concentration; MRSN, Multidrug-Resistant Organism Repository and Surveillance Network; NEG, no flattening of the zone of inhibition toward cefoxitin (30 μg); POS, flattening of the zone of inhibition toward cefoxitin (30 μg); UPEC, uropathogenic *Escherichia coli*.

### Molecular detection of ESBL genes

**FIG. 1.**
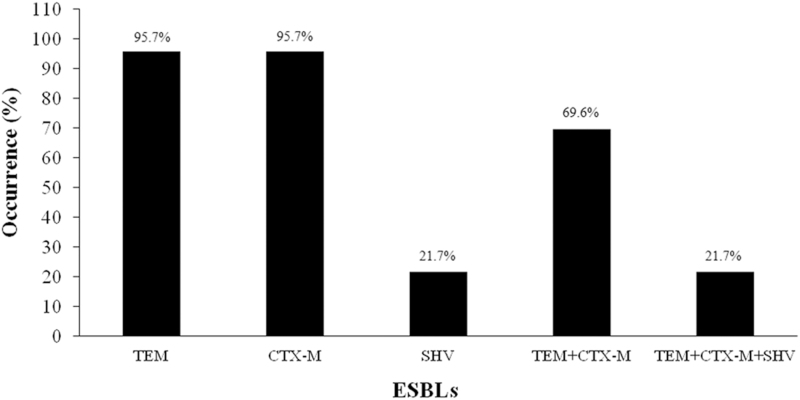
Occurrence of ESBL genes among UPEC isolates indicating the presence of single-gene and multiple-gene combinations of the four ESBL genes: TEM, SHV, and CTX-M among the 23/95 (24.2%) UPEC isolates. CTX-M, cefotaximase-Munich; ESBL, extended-spectrum β-lactamase; SHV, sulfhydryl variable; TEM, temoneira; UPEC, uropathogenic *Escherichia coli.*

All the 23 phenotypically confirmed, ESBL-producing UPEC isolates carried at least one ESBL gene (Table 2). The results indicated that *bla*_TEM_ and *bla*_CTX-M_ were the predominant ESBL genes, each present in 22/23 (95.6%) isolates, followed by *bla*_SHV_ in 5/23 (21.7%) isolates. Five of 23 (21.7%) isolates carried 3 ESBL genes (*bla*_TEM_, *bla*_CTX-M_, and *bla*_SHV_), while 16/23 (69.5%) isolates had both *bla*_CTX-M_ and *bla*_TEM_ genes only (Fig. 1). One of the 23 isolates encoded *bla*_TEM_ and another isolate encoded *bla*_CTX-M_ only (Table 2).

**Table 2.** The Occurrence of the Three Extended-Spectrum β-Lactamase Genes, Temoneira, Sulfhydryl Variable, and Cefotaximase-Munich, Their Phylogenetic Groups, and the Multidrug Resistance Status Among the 23 Uropathogenic *Escherichia coli* Extended-Spectrum β-Lactamase-Producing Isolates

Isolate ID	Resistance status	bla_TEM_	bla_SHV_	bla_CTX-M_	Phylogenetic group
UPEC 1	MDR	+	−	+	B2
UPEC 2	MDR	+	−	+	A
UPEC 3	MDR	+	−	+	B2
UPEC 4	MDR	+	−	−	B2
UPEC 5	MDR	+	−	+	B2
UPEC 6	MDR	+	−	+	D
UPEC 7	MDR	+	−	+	A
UPEC 8	MDR	+	+	+	D
UPEC 9	MDR	+	−	+	B2
UPEC 10	MDR	+	−	+	B2
UPEC 11	MDR	+	−	+	B2
UPEC 12	MDR	+	−	+	B2
UPEC 13	MDR	+	−	+	B2
UPEC 14	MDR	+	+	+	A
UPEC 15	MDR	−	−	+	Ungrouped
UPEC 16	MDR	+	−	+	A
UPEC 17	MDR	+	+	+	F
UPEC 18	MDR	+	+	+	A
UPEC 19	MDR	+	+	+	B_1_
UPEC 20	MDR	+	−	+	B2
UPEC 21	MDR	+	−	+	D
UPEC 22	MDR	+	−	+	B2
UPEC 23	MDR	+	−	+	B2
MRSN/SHV/489100		−	+	−	ND
CTX control		−	−	+	ND
TEM control		+	−	−	ND
Nuclease-free water		−	−	−	ND

Phylogenetic groups are based on the new Clermont quadruplex method (Derakhshandeh *et al.*^30^).

CTX-M, cefotaximase-Munich; MDR, multidrug-resistant; ND, not done; SHV, sulfhydryl variable; TEM, temoneira.

### Multiplex PCR for detection of pAmpC β-lactamase genes

None of the six known pAmpC genes (*bla*_MOX_, *bla*_CIT_, *bla*_DHA_, *bla*_ACC_, *bla*_EBC_, and *bla*_FOX_) were detected by PCR in the cefoxitin-resistant isolates. The *E. coli* CMY-2 (MRSN#570581) strain was used as the positive control, while a DNA-free PCR and nuclease-free water taken through the DNA extraction process were the negative controls.

### Phylogenetic distribution of UPEC isolates

**FIG. 2.**
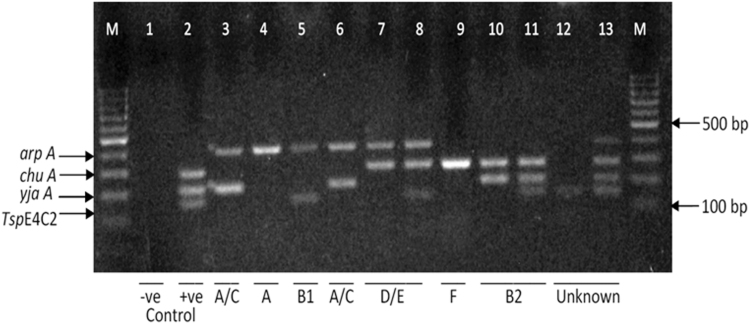
Gel electrophoresis images for the extended quadruplex PCR profile of a few UPEC isolates. The isolates were assigned to a phylo group according to the presence or absence of the following genes in the order *arpA*, *chuA*, *yjaA*, and TspE4C2, where group A is (+ − − −), group A/C is (+ − + −), group B_1_ is (+ − − +), group B2 is (− + + −), group D or E is (+ + − −) or (+ + − +), group F is (− + − −), and other combinations represent unknown groups (− + + +), (− − − +), and (+ + + +). PCR, polymerase chain reaction.

A total of 93 of 95 UPEC isolates were assigned to 5 of the 8 phylogenetic groups using the extended quadruplex PCR method (Fig. 2). Two isolates, 2/95 (2.1%), could not be assigned to a phylogenetic group by this method. The phylogenetic groups assigned to the isolates were A, B_ı_, B2, D, and F (Table 2). Group B2 was the predominant phylogenetic group, 31/95 (32.6%), followed by group D 30/95 (31.6%); group A 18/95 (18.9%); group B_ı_ 11/95 (11.6%); and finally group F 3/95 (3.2%).

## Discussion

Clinical management of UTIs is a major global challenge due to the increasing cases of MDR UPECs. A study on the contribution of UTI to the burden of febrile illnesses in young children in rural Kenya showed a prevalence of 11.9% UTIs.^32^ Another study in Kenya among pregnant women indicated a prevalence of 15.7% UTIs.^6^ UPEC has acquired resistance to the β-lactam antibiotics, commonly used antimicrobial agents for UTI treatment.^21^ Previous studies have reported that the production of ESBL and pAmpC β-lactamases is the leading cause of β-lactam resistance and that the resistance genes are related to phylogenetic grouping.^12,23^ This study aimed to determine the production of ESBL and pAmpC β-lactamases among the archived Kenyan UPEC isolates, screen for the genes associated with resistance, identify their phylogenetic groups, and determine the phylogenetic groups associated with β-lactamase resistance.

The study showed that 24.2% of the UPEC isolates were ESBL producers and multidrug resistant. They were resistant to third-generation cephalosporins, which are often used to treat UTIs. All the 23 ESBL-positive *E. coli* isolates possessed at least 1 of 3 genes found to confer resistance to β-lactams in the Kenyan isolates: *bla*_TEM_, *bla*_CTX-M_, and *bla*_SHV_. These three ESBL genes are prevalent among UPEC isolates in the East African regions as they have been detected in Kenya^15^ and its neighbor, Uganda.^33^ The ESBL genotypes appear ubiquitous among *E. coli* isolates from different sources and are not unique to UPEC isolates.

Although this study showed that 5/23 (21.7%) ESBL-positive isolates were cefoxitin resistant, suggesting AmpC production, none of the known transferable AmpC β-lactamases were detected. These findings were unexpected given previous reports in Kenya^15^ of 10% pAmpC-producing *E. coli* isolated from different clinical samples and 37% AmpC β-lactamases among Enterobacteriaceae from Uganda, with 30 isolates having more than 1 gene coding for AmpC-mediated resistance.^33^ Since the cefoxitin-resistant isolates were not AmpC β-lactamase producers as observed in other studies,^20,26^ it suggests that the isolates in this study may have other enzymatic mechanisms for cefoxitin resistance such as production of different ESBLs or nonenzymatic mechanisms such as decreased porin production,^21^ warranting further investigation.

Determination of *E. coli* phylogenetic groups is of epidemiological importance as several studies indicate that phylogenetic groups could be related to the severity of disease.^34^ These studies demonstrated a relationship between the phylogenetic group and virulence, with more virulent extraintestinal strains belonging to phylogenetic groups B2 and D.^35^ In contrast, most commensals belonged to phylogenetic group A and group B_1_.

In this study, the predominant phylogenetic group was B2 (32.6%), followed by D (31.6%), group A (18.9%), group B_1_ (11.6%), group F, and finally the ungrouped group (2.1%). These were the main phylogenetic groups detected among the isolates studied from diverse parts of the country and could reflect the phylogenetic groups circulating in Kenya. Predominance of groups B2 and D among the Kenyan UPEC isolates indicates virulent strains in the population. The study showed that most ESBL-producing isolates belonged to phylogenetic group B2 and group A (Table 2). The isolates in phylogenetic group B2 were more likely to be ESBL producers compared with other phylogenetic groups (*p* < 0.05) (Table 3). This study has demonstrated a link between the B2 phylogenetic group and penicillin/cephalosporin resistance, combining virulence and antibiotic resistance traits. Tracking of these phylogenetic groups can give an indication of the burden of ESBL producers among UPECs.

**Table 3.** Distribution of Extended-Spectrum β-Lactamase Producers Versus Nonextended-Spectrum β-Lactamase Producers Among the Phylogenetic Groups

Phylogenetic group	ESBL producers,* n *(%)	Non-ESBL producers,* n *(%)	*p*
A			
Positive	5 (27.8)	18 (19.0)	0.5224
Negative	13 (72.2)	77 (81.0)	
B_1_			
Positive	2 (18.2)	22 (20.7)	>0.9999
Negative	9 (81.8)	84 (79.3)	
B2			
Positive	12 (38.7)	11 (14.7)	0.0095
Negative	19 (61.3)	64 (85.3)	
D			
Positive	3 (10.0)	20 (23.5)	0.1826
Negative	27 (90.0)	65 (76.5)	
F			
Positive	1 (33.3)	22 (19.3)	0.4847
Negative	2 (66.7)	92 (80.7)	
Ungrouped			
Positive	1 (50.0)	22 (19.0)	0.3532
Negative	1 (50.0)	94 (81.0)	

Isolates in phylogenetic group B2 were more likely to be ESBL producers compared with other phylogenetic groups (*p* = 0.0095).

The presence of ESBL producers among the commensal groups B_ı_, A, and F shows the potential horizontal transfer of antimicrobial resistance genes from the phylogenetic groups associated with resistance to the typically non-ESBL-producing phylogenetic groups. Isolates of groups C, E, and Clade 1 were not detected in this study. A more extensive UPEC study would be needed to determine if they are absent or just rare in Kenya, as they have been reported in UPEC isolates from Australia and Iran.^31,35^ In our study, two isolates could not be assigned to any of the eight recognized phylogenetic groups using the new extended quadruplex method. This inability to assign isolates to a phylogenetic group confirms the assay's known limitation, the use of primers specific to currently known gene groups, which may not detect minor or novel phylogenetic groups.^31^ New strains could emerge due to large-scale recombination between two or more different phylogenetic groups or by the gain or loss of genes in the *E. coli* genome.^31^ These ungrouped isolates could be other phylotypes of UPEC and are worth investigating in further studies.

## Conclusions

UTIs are the leading cause of outpatient visits, so understanding the epidemiology of UPEC can contribute to better treatment and reduced morbidity of UTIs. This study has determined that ESBL-producing UPEC isolates are present in Kenyan hospitals, with 24.2% of the isolates being resistant to the commonly prescribed cephalosporin drugs for UTIs. This resistance level indicates possible antibiotic selection pressure in Kenyan hospitals and community settings, driving resistance to these widely prescribed drugs. The *bla*_CTX-M_ and *bla*_TEM_ genes predominated in this study, with the coexistence of multiple genes in single isolates indicating increased transmission of genetic determinants and the likely increase of ESBL pathogens in Kenyan hospitals. However, the *AmpC* genes typically associated with cefoxitin resistance were not observed in this study, a possible indication of other cefoxitin-resistant mechanisms that warrant further investigation. The phylogenetic groups B2 and D predominated in this study, while phylogenetic groups B2 and A had the greatest number of ESBL-producing UPECs. Although the study tested only 95 UPEC isolates, the isolates were from diverse regions within Kenya and provided some indication of the resistance patterns and genes among UPEC isolates. The characterization of UPEC phylogenetic groups has contributed to the understanding of the epidemiology of UPEC isolates in Kenya. Identification of the new uncharacterized phylogenetic groups emerging on the landscape opens up new avenues to study these important pathogens using more robust whole-genome approaches.

## Ethical Approval

This study was approved by the KEMRI/Scientific Ethics Review Unit (SERU), (KEMRI/SERU/CCR/0088/3609), and the Walter Reed Army Institute of Research (WRAIR) Institutional Review Board (IRB; WRAIR No. 2089B).
